# Biological and Cognitive Frameworks for a Mental Timeline

**DOI:** 10.3389/fnins.2018.00377

**Published:** 2018-06-11

**Authors:** Catalin V. Buhusi, Sorinel A. Oprisan, Mona Buhusi

**Affiliations:** ^1^Interdisciplinary Program in Neuroscience, Department of Psychology, USTAR BioInnovations Center, Utah State University, Logan, UT, United States; ^2^Department of Physics and Astronomy, College of Charleston, Charleston, SC, United States

**Keywords:** time perception, temporal order, computer simulations, biologically inspired cognitive architectures, brain, neural networks

Animals are molded by natural forces they do not comprehend. To their minds there is no past and no future… only the everlasting present of a single generation, its trails in the forest, its hidden pathways in the air and in the sea… There is nothing in the Universe more alone than Man. He has entered into the strange world of history…

– *Loren Eiseley (1960)*

## Introduction

Historians like to order long-gone events in time. When events correlate with years—*numbers*—events seem to follow a clear time line, but when their order is unclear, historians order events using extra information from folklore, writings, artifacts, and cultural habits. Here we ask the following question: How does the brain, at a neuromechanistic level, order events on a mental time line? This question is relevant to many neuroscience paradigms such as rate calculation, planning, and decision making, processes that crucially depend on the order of events. For example, episodic memory incorporates order and duration of the events in the episode (Tulving and Donaldson, 1972; Eichenbaum, 2017). Events and their features (order, duration, content etc.) are stored in memory and recalled when needed. But how is the order of events assessed when events are recalled from memory to be placed on the timeline? To address this question, we discuss several classes of models of timing and time perception, and their capability of ordering events in time. Because the mental time includes all durations, our discussion will freely mix time scales: milliseconds, seconds, hours, days. Moreover, here we do not discuss in depth the scalar property—the increase in timing errors with the criterion time—because almost all models of timing can reproduce the scalar property, making it a weak criterion for selecting among these models.

## Cognitive frameworks

Cognitive models of time perception readily implement the “mental timeline” paradigm even when they use an internal representation of time which is very much not timeline-like.

### Pacemaker-accumulator models

**Figure 1:**
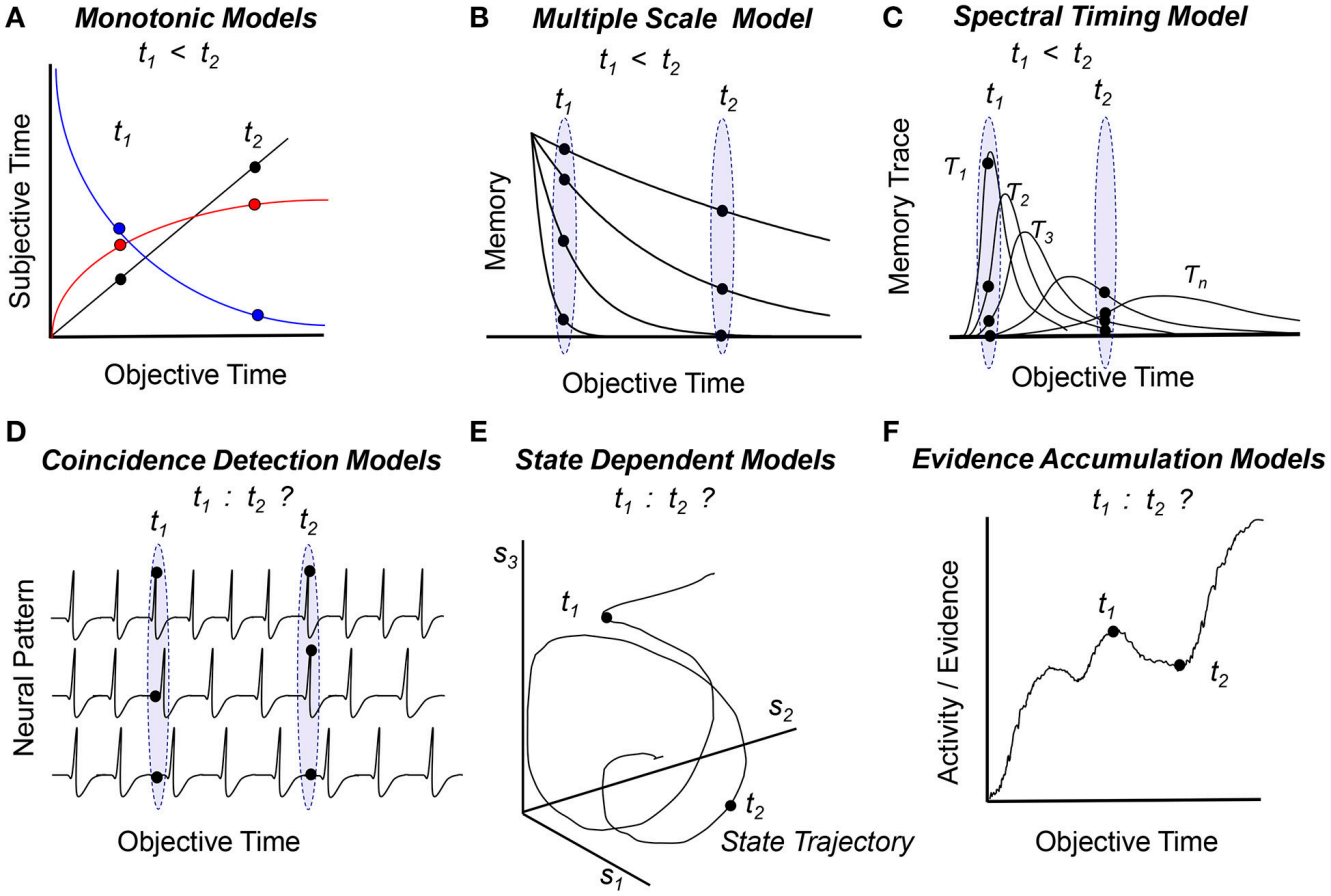
Temporal order within the framework of cognitive **(A–C)** and biological **(D–F)** models of timing and time perception. Panels indicate how models assess temporal order of two events at times *t*_1_ and *t*_2_ (see text for details) **(A)** Subjective time is a monotonic function of objective time (linear—black, logarithmic—red, or exponentially decaying—blue), such that the objective order of events can be inferred from the subjective representation of time, *t*_1_ < *t*_2_. **(B)** Multiple monotonic (exponentially-decaying) memory traces can convey temporal order. **(C)** Multiple non-monotonic traces that evolve at different speeds can also convey temporal order. **(D)** An internal representation of time based on patterns of firing neurons cannot in itself convey temporal order, as patterns have no intrinsic order. **(E)** Rather than being a *coordinate*, time could be considered a *parameter* of a system that follows a trajectory in a state-based coordinate system {*s*_*1*_, *s*_*2*_, …, *s*_*n*_}. Such systems can equally follow the same state trajectory toward the future or toward the past, thus, they have difficulty ordering events in time. **(F)** The pattern of activity of a population of neurons varies in time as the model accumulates evidence; evidence / activity / patterns correlate with time but are not solely representing time. Biological models **(D–F)** need extra assumptions / transformations / information to map activity / states / evidence / patterns to order of events; such information may be provided by chemical, electrical, and circuit level constraints rather than time itself.

The *Internal Clock Model* (Treisman, 1963) and *Scalar Expectancy Theory* (Gibbon, 1977) assume that time is represented subjectively by the number of accumulated pacemaker pulses (black line in Figure 1A). An alternative monotonic function proposed to link the objective and subjective representations of time is the logarithmic function (red curve in Figure 1A) (for a discussion see Staddon and Higa, 1999). Because the subjective time is a monotonically increasing function of the objective time, and because events are stored in memory as numbers which carry intrinsic ordinal information, in these models the order of events is naturally preserved in memory. Moreover, in the *Scalar Expectancy Theory* subjects can also manipulate these numbers, such that the duration of the interval between two events is readily available as *t*_*2*_ – *t*_*1*_ (Church, 1984; Gibbon and Church, 1990). Therefore these models readily embody the “mental timeline” paradigm (Church, 1978).

### Pacemaker-free models

To tell time, historians use radiocarbon-dating, a method in which the age of an object is estimated based on C^14^ radioactive decay. Interestingly, the brain's circadian system uses a similar system to tell the time of day based on protein degradation (Golombek and Rosenstein, 2010). Such timing mechanisms motivated a distinct set of cognitive models which rely on monotonically decaying functions, such as the *Adaptive Decay Model* (Dragoi et al., 2003) (the blue curve in Figure 1A) and the *Multiple Time Scales Model* (Staddon and Higa, 1999) (Figure 1B). Because time is coded by (one or many) monotonically decaying functions, these models can order events in time simply by comparing the numbers/patterns corresponding to the events.

### Distributed models

While *Pacemaker-Accumulator Models* represent time by storing only one piece of information (the number of pulses), real physical systems, e.g., mechanical wristwatches, keep track of time using multiple mechanisms working at different time scales, e.g., an hour-hand, a minute-hand, and a seconds-hand. This motivated the development of cognitive models of timing using multiple non-monotonic distributed processes which evolve at different speeds (scales), such as the *Spectral Timing Model* (Grossberg and Schmajuk, 1989; Buhusi and Schmajuk, 1999; Figure 1C). In this model events are represented by distinct non-monotonic patterns of memory traces. Because traces evolve at different speeds, they can be correctly ordered on a time line, *t*_*1*_ < *t*_*2*_, in a manner similar to comparing the pattern of the hands on the wristwatch with a desired time, despite using an internal representation of time which is very much not timeline-like.

## Biological frameworks

While cognitive models readily order events on a time line, biologically-inspired models have difficulties ordering events because they process and store events in memory as neural patterns, which lack intrinsic ordinal value.

### Coincidence detection models

A class of biologically-inspired timing models assume that time is represented by the coincidental activation of multiple neuronal inputs. For example, in the *Coincidence Detection Model* (Miall, 1989) and in the *Striatal Beat Frequency Model* (Matell and Meck, 2004; Buhusi and Meck, 2005), timing is coded by the pattern of multiple neuronal oscillators (Figure 1D). Supplemental assumptions are used to map the models onto the brain: For example, the *Striatal Beat Frequency Model* ascribes a role for detecting event durations to medium spiny neurons within the dorsal striatum which become entrained to fire in response to oscillating, coincident cortical inputs. Interestingly, no extra assumptions are needed to describe the scalar property, which emerges in the *Striatal Beat Frequency Model* due to neural noise (Buhusi and Oprisan, 2013; Oprisan and Buhusi, 2013a,b, 2014). However, when events are recalled, these models have difficulty assessing the order of events, as there is no predetermined order of neural patterns. These models need extra information to order of events in time, which may be provided by circuit level constraints, such as the unidirectionality of action potentials.

### State dependent models

Another way real physical systems code for time is in their (distributed) state. For example, winter is different from summer in all the changes in foliage, temperature, precipitation etc. Similarly, in the *State Dependent Timing Model* (Buonomano and Maass, 2009) the system follows a trajectory along which states (events) are coded in time (Figure 1E). When events (states) are recalled from memory, the model has difficulty ordering events, pretty much like one has difficulty saying whether summer follows winter or rather winter follows summer. In fact, state dependent models can follow the same trajectory “forward” in time, as well as “backward” in time, since time is a *parameter* rather than a *coordinate* in these models. Thus, state dependent models are physically- and biologically-inspired, but need extra information to implement a unidirectional timeline. Extra information to order events in time may be provided by chemical reactions, as not all chemical reactions are bi-directional; this type of information may limit the trajectory of the system, and provide a sense of order.

### Evidence accumulation models

Another set of biologically-inspired timing models take advantage of the observation that during an interval the activity of specific populations of neurons largely increases as shown in Figure 1F (Leon and Shadlen, 2003; Mita et al., 2009; Xu et al., 2014). This (non-linear) increase in activity was suggested to reflect neuronal integration (Simen et al., 2011), drift-diffusion processes (Luzardo et al., 2013), or accumulation of evidence (Leon and Shadlen, 2003). Notably, the similarity between *Evidence Accumulation Models* (Figure 1E) and *Pacemaker-Accumulator Models* (Figure 1A) is misleading. The latter assume that time is stored in memory as (ordered) *numbers*, while the former store in memory the *patterns of neural activation/evidence*, supposedly devoid of order. Not only *Evidence Accumulation Models* work with patterns, but the nature of the information manipulated/stored (activation or evidence) is different than in *Pacemaker-Accumulator Models* (pulses or numbers). *Evidence Accumulation Models* can compare events in terms of evidence/patterns of activation, but not necessarily in time. It would require an extra assumption (transformation) to map activation or evidence into order of events. For example one could *assume* that more activity/evidence represents a later event, but whether the brain follows this assumption or not it is not known at this time.

The brain seems to need extra sources of information—*at the chemical, electrical, circuit level—*than time itself to order memory patterns in a time line. This idea is consistent with recent experimental evidence suggesting that time and order of events are coded by different processes in the brain (D'argembeau et al., 2015).

## Conclusions

While cognitive models of timing and time perception seem readily equipped to represent order of events on a mental time line, they do not do so in a realistic manner. Meantime, biologically-plausible models face specific challenges when ordering events into “past,” “present” and “future.” However, as an electro-chemical network, the brain may impose its own chemical, electrical, and circuit level constraints that could provide a sense of order other than time itself. Either way, it seems that more work needs to be done at both ends of the spectrum of theories, toward an explanation of the mental time line that is biologically realistic. One major objective of a science of the mental time should be to incorporate order—and not necessarily *number*—into biologically-inspired theories of timekeeping to understand how the brain represents time and order on the mental timeline. We speculate that the apparent mental time line readily embodied by *Scalar Expectancy Theory* is represented in the brain in a distributed manner—much as in the *Spectral Timing Model* (Grossberg and Schmajuk, 1989; Buhusi and Schmajuk, 1999)—at multiple brain regions, including striatum (Bakhurin et al., 2017), amygdala (Dallérac et al., 2017), hippocampus (Eichenbaum, 2014), lateral intra-parietal sulcus (Jazayeri and Shadlen, 2015), insula (Wittmann et al., 2010), and even visual areas (Shuler, 2016). In turn, these brain regions may use their own system to code the passage of time, possibly similar to *Coincidence Detection Models* (Matell and Meck, 2004; Buhusi and Meck, 2005), *State Dependent Models* (Buonomano and Maass, 2009), or *Evidence Accumulation Models* (Leon and Shadlen, 2003; Simen et al., 2011; Luzardo et al., 2013) discussed above. In this scenario, timing is a process distributed at multiple levels, molecular, local circuits, and brain-wide circuits (Buhusi et al., 2016) that generates a linear timeline at the behavioral level from multiple non-linear local timelines. Future research should differentiate and integrate a “sense of time passage” with “a sense of order” of events and their biological substrates that enable the (re)construction of a mental time line.

## Author contributions

All authors listed have made a substantial, direct and intellectual contribution to the work, and approved it for publication.

### Conflict of interest statement

The authors declare that the research was conducted in the absence of any commercial or financial relationships that could be construed as a potential conflict of interest.
